# Human CD21^lo^T-bet^+^ B cells: Not as easy as “ABC”!

**DOI:** 10.70962/jhi.20260023

**Published:** 2026-04-16

**Authors:** Stuart G. Tangye

**Affiliations:** 1 Garvan Institute of Medical Research, Darlinghurst, Australia; 2 School of Clinical Medicine, Faculty of Medicine and Health, University of New South Wales Sydney, Sydney, Australia

## Abstract

B cells contribute to many facets of adaptive immunity, including key roles as Ag-presenting cells, cytokine-producing cells, and plasma cells secreting protective antibodies. However, B cell functional dysregulation can result in myriad immune dyscrasias, such as immunodeficiency, chronic infection, autoimmunity, allergy, and malignancy. Thus, it is critical to understand fundamental aspects of human B cell differentiation and effector function. A B cell subset that has attracted much attention over the past 2 decades is a population known by many identities—CD21^lo^, atypical memory, CD27^neg^IgD^neg^, age-associated, exhausted—and associated with many diseases, especially humoral immune dysregulation. However, these cells likely also contribute to humoral immunity in the setting of vaccination and natural infection. This Review tries to provide an overview of the discoveries, origins, and complexities of CD21^lo^ B cells, and how studying inborn errors of immunity can provide a unique window to understand the molecular requirements for generating these cells, as well as mechanisms underpinning function in health and disease.

## Teaser

This Review provides an overview of the discoveries, origins, and complexities of CD21^lo^ B cells, and how studying inborn errors of immunity can provide a unique window to understand the molecular requirements for generating these cells, as well as mechanisms underpinning their function in health and disease.

## Introduction

### B cell development, differentiation, and function

B cells have myriad functions during immune homeostasis and immune responses, including lymphoid tissue organogenesis, antigen (Ag) presentation, CD4^+^ T cell stimulation, and production of neutralizing antibodies (Abs) (1). These diverse functions of B cells reflect their nature as shapeshifters. Thus, B cells acquire different and/or distinct states, functions, and phenotypes, along with molecular (re)programming, as they become activated and differentiate in response to external stimuli provided by foreign Ag and the microenvironment within sites of immune activation.

**Figure 1.**
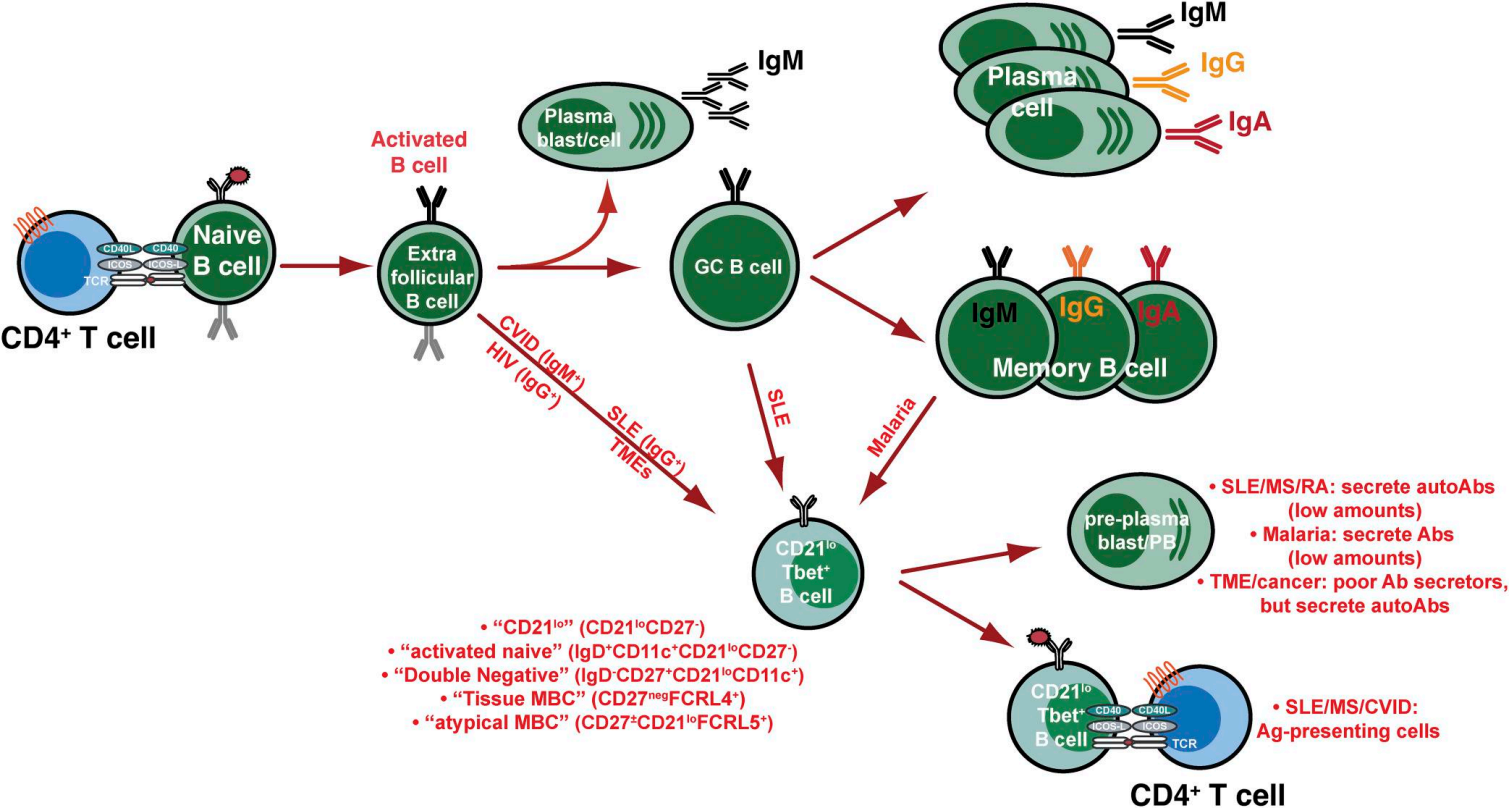
**Human B cell differentiation pathways of multiple effector subsets.** When naïve B cells receive signals via the BCR or T cell help in the form of CD40L and cytokines, they can either develop into short-lived plasmablasts secreting predominantly IgM, or form GCs, which give rise to long-lived MBCs and PCs capable of secreting IgM, IgG, and IgA. Activated B cells can also acquire expression of the transcription factor T-bet to differentiate into CD11c^+^CD21^lo^T-bet^+^ B cells. CD11c^+^CD21^lo^T-bet^+^ B cells can likely arise from naïve/extrafollicular, GC, or MBCs. Several subsets of CD11c^+^CD21^lo^T-bet^+^ B cells have been identified based on distinct phenotypes and disease states, as indicated. Whether these subsets represent precursor/progeny remains to be determined. CD11c^+^CD21^lo^T-bet^+^ B cells are enriched for production of autoAbs and can also present autoAg to CD4^+^ T cells to initiate activation of other autoreactive B cells. CVID: common variable deficiency; SLE: systemic lupus erythematosus; HIV: human immunodeficiency virus; MS: multiple sclerosis; RA: rheumatoid arthritis; TME: tumor microenvironment.

Many studies performed in humans, mice, and animal models have identified molecular, biochemical, and cellular requirements for generating distinct B cell subsets during humoral immune responses and the establishment of B cell memory (2, 3). These studies not only revealed what is needed to achieve effective humoral immunity but also highlighted that B cell differentiation is stringently regulated as perturbations to this process can result in a constellation of immune dyscrasias including recurrent infection, autoimmunity, or allergy. Thus, we have a clear understanding of the origins, functions, and regulation of naïve B cells, germinal center (GC) B cells, memory B cells (MBCs), and plasma cells (PCs), as well as the relationships between these B cell subsets (2, 3, 4) (Fig. 1).

Over the past 20^+^ years, an enigmatic B cell subset—variably known as “activated naïve,” “atypical memory,” “tissue-like memory,” “exhausted B cells,” “double-negative (CD27^^−^^IgD^−^, DN) B cells,” or “age-associated B cells” (ABCs)—has moved into the spotlight. This B cell population is highly heterogeneous, comprising several subsets that can be defined by overlapping but often distinct phenotypes, transcriptomes, and functions. These B cells, which I will generally refer to as CD21^lo^T-bet^+^ B cells, appear chameleon in nature, with multiple—and often opposing—functions depending on the immune context (Fig. 1). There have been many excellent reviews on this topic recently, which include current ideas and concepts drawn from a plethora of studies from humans, mice, and models of human disease (5, 6, 7, 8, 9). I refer readers to these reviews for a deep dive into and a granular analysis of the many flavors of this B cell subset, especially for studies in mice. In this Review, I will strive to provide a history of the discovery of human CD21^lo^T-bet^+^ B cells, define the nature of these cells in settings of health and many disease settings, and discuss how inborn errors of immunity (IEIs) offer a terrific opportunity to establish the pathways required for the ontogeny, differentiation, and physiological function(s) of these cells in healthy humans, their dysfunction in human immune dyscrasias, and whether targeting these cells would have therapeutic benefits.

## CD21^lo^ B cells

### In the beginning: The original discovery of human CD21^lo^ B cells

**Figure 2.**
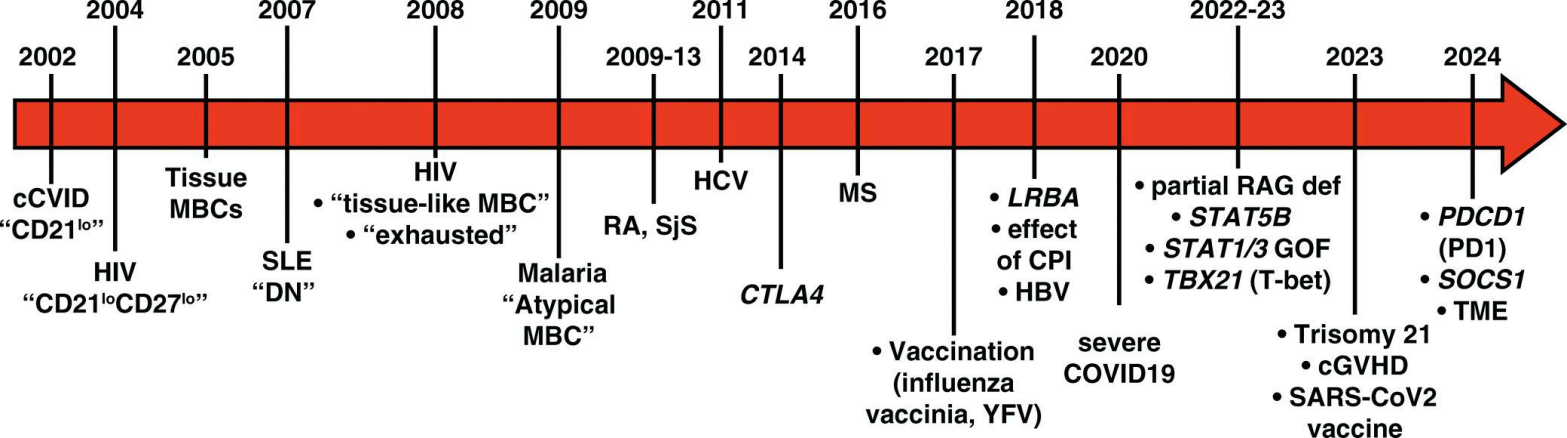
**Timeline of key events relating to human CD21** ^**lo**^ **B cells.** MBC: memory B cells; cCVID: complex CVID; MS: multiple sclerosis; DN: double negative; SLE: systemic lupus erythematosus; RA: rheumatoid arthritis; SjS: Sjogren’s syndrome; YFV: yellow fever vaccine; HBV: hepatitis B virus; HCV: hepatitis C virus; HIV: human immunodeficiency virus; cGVHD: chronic graft-vs.-host disease; TME: tumor microenvironment.

**Figure 3.**
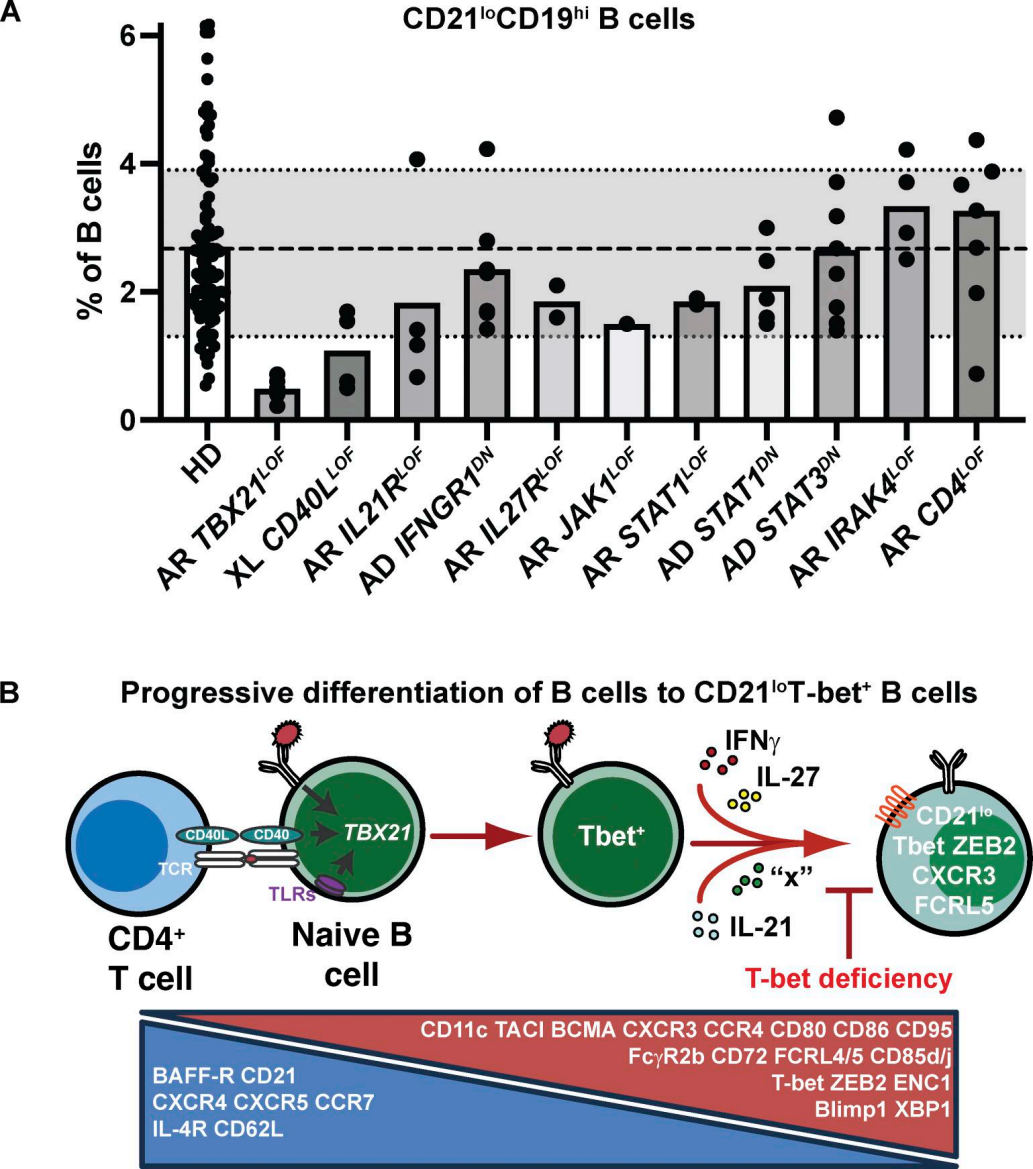
**CD21** ^**lo**^ **B cells in IEIs. (A) **CD21^lo^CD19^hi^ B cells were quantified in HDs (*n* = 94), as well as patients with the indicated IEI. The dashed line (-----) represents the mean of CD21^lo^CD19^hi^ B cells in HDs; the dotted lines (_……_) represent 1 standard error of the mean. AD: autosomal dominant; AR: autosomal recessive; XL: X-linked. Data for AR *TBX21* deficiency are derived from a single patient but from multiple blood samples. Only one patient was available to test for AR JAK1 and AR STAT1 deficiency. For all other IEIs, multiple patients were tested. These data have previously been published in studies from the Tangye lab (16, 17, 18, 19, 20). **(B)** Scheme of differentiation of naïve B cells into CD21^lo^T-bet^+^ B cells. Integration of signals through the BCR in combination with CD40 or TLRs induces expression of T-bet in B cells. T-bet expression is further increased following exposure to cytokines, such as IFNγ, IL-27, or IL-21 (possibly other cytokines, “x”). Cytokine-induced T-bet upregulation is required for further maturation of T-bet^+^ B cells, evidenced by acquisition of canonical surface receptors such as CXCR3, FCRL4/5, and CD11c. This second phase of CD21^lo^T-bet^+^ B cell generation is abolished by T-bet deficiency (19).

In 2002, Klaus Warnatz and colleagues described a novel human B cell subset characterized by the reduced expression of the complement receptor CD21, termed “CD21^lo^ B cells” (Figs. 1 and 2). CD21^lo^ B cells comprise a minor subset of peripheral blood B cells in healthy individuals (∼1–5%, Fig. 3 A) but were increased (>20% of all B cells) in a subgroup of CVID patients who developed non-infectious immune complications such as organomegaly, anemia, cytopenias, and autoantibodies (autoAbs), but also exhibited poor responses following vaccination (Table 1) (10, 11). This subset of CVID patients will be referred to as “complex CVID (cCVID).” Subsequent studies from the Warnatz lab established that CD21^lo^ B cells from cCVID patients were phenotypically distinct from conventional CD21^+^ B cells; i.e., CD21^lo^ B cells were large cells expressing lower levels of CD24, CD27, and CD38 (in addition to CD21) and higher levels of CD19, CD20, and IgM than CD21^+^ B cells (12) (Table 1). Interestingly, CD21^lo^CD38^lo^ B cells can be detected in all major subsets of human peripheral blood B cells, i.e., naïve, and IgM^+^ and class-switched MBCs, indicating that CD21^lo^ B cells are not strictly MBCs and are likely to arise from various precursor B cell populations (Fig. 1) (13). Transcriptomic analyses not only extended the phenotypic differences between naïve and cCVID CD21^lo^ B cells, with the latter expressing high levels of transcripts encoding immunoregulatory/inhibitory receptors (*CD22*, *CD32 *[*FCGR2B*], *CD72*, *TNFRSF13B* [TACI]), inflammatory chemokine receptors (*CXCR3*), adhesion molecules (*ITGAX* [CD11c], *ITGB7* [CD49d]), and various transcription factors (*SOX4*, *TBX21* [encoding T-bet], *TOX*) but also reduced mRNA levels encoding molecules associated with homeostatic lymphocyte trafficking (*CXCR4*, *CXCR5*, *CCR7*, *CD62L*) (12, 14, 15) (Table 1). CD21^lo^ B cells had undergone greater proliferation in vivo than naïve B cells from both cCVID patients and healthy donors (HDs). However, levels of somatic hypermutation (SHM) in Ig V region genes expressed by cCVID CD21^lo^ B cells were only modestly greater than naïve B cells, and significantly less than MBCs (12), suggesting these cells likely arise from activated naïve B cells rather than GC reactions (Fig. 1 and Table 1). Functional studies indicated that cCVID CD21^lo^ B cells had impaired responses in vitro following engagement of the BCR, CD40, or TLRs (Table 1) (10, 11, 12, 14).

**Table 1.** Many types of human CD21^lo^ B cells identified in disease and healthy states

Disease/tissue	Defining phenotype	Ig isotype	SHM	In vitro responses	Disease correlates	TFs	Additional phenotypic markers
CVID	CD21^lo^	Mostly IgM	Low (∼naïve)	↓ proliferation ↑ Ig secretion	• Serum IFNγ, IFNγ-induced soluble factors (CXCL10) • cTfh1 cells	↑ SOX4 TBX21 (T-bet) TOX	↑ CD19 IgM CD95 CXCR3 *CD22CD32 (FCGR2B) CD72 TNFRSF13B* (TACI) *ITGAX* (CD11c) *ITGB7* (CD49d) ↓ CD38 CD24 CD27 *CXCR4CXCR5CCR7CD62L*
Tissue-based MBC	FCRL4^+^CD27^-^	Mostly IgG, IgA	Similar to MBC	↓ proliferation ↑ Ig secretion	NA	↑ RUNX1 SOX5	↑ CD20 CXCR3 CD32 (FcRγIIB) CD80 CD86 CD11c CD95 RANKL CD40 *CCR1 CCR5 CCR6* ↓ CD21 CD31 *CXCR4 CXCR5 CCR7*
HIV	CD21^lo^CD27^−^ “Tissue-like/exhausted MBC”	IgG (G1, G3), IgA, some IgM	Intermediate between naïve and MBC	↓ proliferation ↓ Ig secretion	• IFNγ-induced gene signature	↑ T-bet, RUNX3 SOX5 TOX1	↑ FCRL4 CD72 CXCR3 CCR6 CD11c CD85j CD22 LAIR-1 TACI BCMA CD95 ↓ CXCR4 CXCR5 CD62L CCR7 BAFF-R
Malaria	CD21^lo^CD27^lo^ Atypical MBC	IgG > IgM	Similar to MBC Derived from MBC	↓ proliferation ↓ Ig secretion	• Serum IFNγ	↑ T-bet ↓BACH	↑ FCRL5 CXCR3 CD11c CD85j CD19 CD20 CD22 FcγRIIb ↓ CD27 CXCR4 CXCR5 CD62L CCR7
HCV	CD21^lo^	​	Similar to MBC	↓ proliferation	​	↑ SOX5 ZEB2 ↓BACH2	↑ CD11c FCRL4 CD19 CD20 CD22 CD72 CD95
cHBV infection (including HBsAg-specific B cells)	CD21^lo^ CD27^±^	Mostly unswitched	ND	↓ Ca^2+^ flux, BCR signaling ↓ Ig secretion ↓ cytokine production ↑ apoptosis	• Low HBV-specific IgG	↑ T-bet	↑ CD11c CXCR3 FCRL5 CD22 BTLA PD1 FcγRIIb ↓ CXCR5 CD80
SLE	•IgD^−^CD27^−^ (DN) •Activated B cells • CD19^hi^CD20^hi^ • CD11c^+^ • CD24^−^CD20^hi^	IgG	> naïve < MBC	↑ autoAb secretion ↑ apoptosis	• Disease severity, serum autoAbs, renal involvement, lupus nephritis	↑ T-bet ZEB2 BCL6 IRF4 Blimp1^±^ XBP1	**• DN2**: CXCR5^neg^ CD19^hi^ CD21^lo^ CD11c^+^ **• CD11c+**: ↑ CD19 CD20, CD32 BAFF-R CD95 IL21R FCRL5 CD72 CXCR3 CCR9 SYK CD79B TLR9 CD80 CD86; TACI^int^, ↓ CXCR4 CXCR5 CCR7 **• CD24**^**−**^**CD20**^**hi**^: CD21^lo^ CD11^hi^ CD95^hi^ CD27^±^ SLAMF7^hi^; ↑ *PDCD1 FCRL3 ILIRB1 ILIRB2*
Other AI (RA, SjS, GVHD, T1D)	CD21^lo^	IgM, IgG, IgA	Low (T1D)	↓ proliferation	• Disease severity, autoAbs	↑ T-bet SOX4 SOX5 TOX *ZEB2*	↑ CD19 CD22 CD86 CD58 CD11c CD72 CD32 CD85j CD85d Fas RANKL (RA) *ITGAX FCRL5* ↓ CD40 BAFF-R IL-4R CCR7 CXCR4 CD44 CD62L CD27^int^
Trisomy 21	CD11c^+^	IgM, IgG, IgA	Reduced	Intact Ig secretion	• Inflammatory cytokines (IL-6) • cTfh1/17 cells • Circulating PCs	↑ T-bet	↑ CD86 CD95 CXCR3 CCR4 ↓ CD21 CXCR5 CCR7
Cancer	scRNA-seq CD21^−^CD27^−^ DN	​	Low	↓ BCR response ↓ plasmablast generation but autoAb secretion	• Poor outcomes • Worse response to ICI blockade in some cancers	↑ *TBX21* (T-bet) *ZEB2 PRDM1IRF4XBP1TOXTOX2* ↓*IRF8*	↑ *FCRL4 FCRL5 CD32A CD32B CD72 ITGAX* (CD11c) *PDCD1* (PD-1) *CD80CD86 SYK TLR7 TLR9 DUSP4* CD85j LAIR1 ↓ *CR2* (CD21) *CD27 CD38*
Vaccine recall	CD21^lo^CD27^+^ CD21^lo^CD27^−^CD11c^+^CD21^lo^	​	​	↓ Ig secretion	• Vaccine-specific IgG • cTfh1 cells	↑ T-bet Blimp1 XBP1	↑ CD85j CD11c FCRL5 Ki67 CD80 CD95 BCMA ↓ CD38 CXCR4 CXCR5
HDs	CD21^lo^CD27^-^	**FCRL5** ^**−**^: IgM, IgD **FCRL5**^**+**^: IgM, IgG	**FCRL5** ^**−**^: low **FCRL5**^**+**^: increased but <MBCs	↓ proliferation compared with naïve B, but FCRL5^−^ > FCRL5^+^ ↑ apoptosis	​	**FCRL5** ^**+**^: ↑ *TOX TOX2 TBX21 SOX5 BCL6* ↓ *FOXP1 EGR1*	**FCRL5** ^**-**^: CD32^+^ LAIR1^+^ PD1^+^ CCR7^lo^ CD62L^lo^ CXCR3^hi^ CXCR4^lo^ CXCR5^lo^ BAFF-R^lo^ CD11c^dim^ CD20^++^ CD86^+^ CD95^+^ TACI^dim^ **FCRL5**^**+**^: CD22^hi^ CD32^hi^ LAIR1^hi^ PD1^hi^ CCR7^lo^ CD62L^lo^ CXCR3^hi^ CXCR4^neg^ CXCR5^neg^ BAFF-R^lo^ CD11c^hi^ CD20^++^ CD86^hi^ CD95^hi^ TACI^+^

CVID, common variable immune deficiency; MBCs, memory B cells; cTfh cell, circulating T follicular helper cell; SLE, systemic lupus erythematosus; RA: rheumatoid arthritis; SjS: Sjogren’s syndrome; HBV, hepatitis B virus; HBsAg, hepatitis B surface Ag; HCV, hepatitis C virus; T1D, type 1 diabetes.

To understand possible mechanisms underlying CD21^lo^ B cell expansion in cCVID patients, several studies investigated biomarkers as correlates of these cells. Memory CD4^+^ and circulating T follicular helper (cTfh) cells in blood and lymphoid organs of cCVID patients were skewed to a T helper 1 (Th1) fate (CXCR3^+^CCR6^−^, ↑IFNγ^+^) compared with CD4^+^ T cells from non-complex CVID patients and HDs (Table 1) (21, 22). Interestingly, cCVID CD21^lo^ B cells exhibited an IFNγ gene signature, suggesting overproduction of IFNγ in cCVID—probably by Th1-skewed Tfh cells—directly impacted the generation and/or function of CD21^lo^ B cells (22). A link between IFNγ, CD21^lo^ B cells, and immune dysregulation was strengthened by proteomic analysis of serum inflammatory cytokines/chemokines that could cluster CVID patients into two major groups—one with greater incidence of non-infectious complications and increased frequencies of cTfh cells and CD21^lo^ B cells (thus corresponding to cCVID) compared with the other group of CVID patients (23). The predominant proteomic signature of cCVID was defined by IFNγ and IFNγ-induced molecules such as the CXCR3-binding chemokines CXCL9, CXCL10, and CXCL11 (23). Strikingly, proportions of CD21^lo^CXCR3^+^T-bet^+^ B cells in cCVID correlated with serum CXCL10 levels, as well as severity of immune dysregulation in cCVID, while CXCL9, CXCL10, and CXCL11 all correlated with proportions of cTfh-type cells (Table 1) (15, 23). Thus, dysregulated production of IFNγ by CD4^+^ T cells may directly contribute to the expansion of CD21^lo^T-bet^+^ B cells in cCVID.

### FCRL4^+^ tissue–based MBCs

One of the most fundamental advances in basic, clinical, and translational immunology was the discovery of B cells by Max Cooper in 1965 (24). For this reason, it is quite apt that Max Cooper contributed significantly to establishing key features of CD21^lo^ B cells, albeit known by a different name (a recurring theme in this field!). In 2005, Ehrhardt and colleagues described a novel population of B cells anatomically restricted to human tonsils and defined by expression of the inhibitory molecule FCRH4 (renamed as FCRL4) (25) (Table 1, Fig. 1, and Fig. 2). FCRL4^+^ B cells had undergone Ig class switching and SHM and were large cells expressing numerous activation and costimulatory receptors (CD69, CD80, CD86) (25). Thus, FCRL4^+^ B cells exhibited many features of MBCs (25), but lacked the canonical human MBC marker CD27 (26, 27). Further phenotypic, molecular, and transcriptomic analysis revealed FCRL4^+^ B cells had higher expression of CD11c, CD20, CD32 (FcRγIIB), CD40, CD95, *CCR1*, *CCR5*, *CCR6*, *CXCR3*, and RANK ligand (RANKL) and the transcription factors *RUNX2* and *SOX5*, but lower expression of CD21, CD31, *CXCR4*, *CXCR5*, and *CCR7* than FCRL4^−^ B cells (Table 1) (25, 28). There was also evidence of greater proliferation in vivo of FCRL4^+^ versus FCRL4^−^ B cells (28). Intriguingly, FCRL4^+^ B cells exhibited poor BCR-induced proliferation in vitro but could produce substantial amounts of Ig in response to T-dependent signals (25). These studies identified a novel population of CD27^−^ MBCs compartmentalized in human tonsils—thus termed tissue-based MBCs—that may be important in humoral immunity at epithelial sites (Table 1) (25, 28).

### Chronic pathogen infection and CD21^lo^ B cells

#### HIV

In the early 2000s, Susan Moir and Tony Fauci began assessing B cell dysregulation in HIV infection. An early finding was that a population with reduced *CR2* mRNA and surface CD21 expression accumulated in individuals with high viral loads (29) (Fig. 2). These CD21^lo^ B cells underwent limited proliferation in response to various stimuli including BCR engagement, but secreted substantial amounts of IgG, and exhibited a plasmacytoid-type morphology (Table 1) (29). Antiretroviral therapies (ART) reduced viral loads, as well as CD21^lo^ B cell proportions (29, 30). Interestingly, CD21^lo^ B cells were higher in people with chronic HIV infection compared with those recently infected, yet the contraction of this population was comparable in response to ART (30). This suggests expansion of CD21^lo^ B cells is dependent on ongoing viral replication. Subsequent studies reported that CD21^lo^ B cells in HIV viremic individuals upregulated expression of TACI, BCMA, CD95, CXCR3, *TBX21*/T-bet, *RUNX1*, *SOX5*, and *TOX1*, downregulated BAFF-R, were enriched for transcripts encoding IFN-stimulated genes, and were more prone to apoptosis than CD21^+^ B cells (31, 32) (Table 1). A subset of CD21^lo^ B cells in HIV viremic individuals expressed FCRL4 (33), as well as other inhibitory (CD22, CD85j, LAIR-1) and inflammatory tissue-homing trafficking receptors (CXCR3, CCR6, CD11c) (Table 1) (33). Intriguingly, CD21^lo^ B cells in infected individuals were enriched for HIV-specific B cells (32, 33, 34), while B cells specific for other pathogens resembled conventional MBCs (32). Based on similarities to tissue-based MBCs described in human tonsils by Max Cooper (25, 28), as well as impaired responses, CD21^lo^ B cells in HIV viremia were termed “tissue-like MBCs” and were proposed to be exhausted B cells (33) (Figs. 1 and 2; and Table 1). CD21^lo^CD19^hi^ B cells could also be detected in lymph nodes of HIV-infected individuals (35). These B cells were phenotypically similar to those expanded in peripheral blood of HIV-infected individuals and were also enriched for HIV-specific cells despite having undergone limited SHM (35). Thus, studies of HIV infection identified a B cell population that may contribute to humoral immune dysfunction/dysregulation, including poor HIV-specific IgG responses, in infected individuals but are responsive to ART (29, 31, 33). Dysfunction of these B cells in HIV may be regulated by inhibitory receptors. Indeed, siRNA-mediated downregulation of *FCRL4* and other inhibitory receptors on CD21^lo^ B cells increased BCR-induced proliferation and differentiation to HIV-specific Ab-secreting cells (36). Thus, modulating expression/function of individual or combinations of inhibitory receptors on CD21^lo^ B cells may restore B cell function in conditions where these cells are overrepresented.

#### Malaria

CD21^lo^CD27^−^ B cells—termed atypical MBCs—were increased in Malian children and adults infected with the malaria-causing parasite *Plasmodium falciparum* compared with healthy U.S. adults (37, 38), as well as in Europeans experiencing initial or subsequent *P. falciparum* infection (39) (Fig. 2 and Table 1). These atypical MBCs expressed T-bet, were predominantly IgG^+^ with an enrichment for IgG3^+^ cells (37, 38, 39, 40), and exhibited an in vivo proliferation and SHM load similar to conventional MBCs (38). Proportions of CD21^lo^CD27^−^ and CD21^lo^CD27^+^ B cells were higher in re-infected individuals compared with people experiencing primary infection (39), suggesting expansion from preexisting *P. falciparum*–specific MBCs (Fig. 1). Malaria-associated atypical MBCs expressed higher levels of CD11c, CXCR3, CD22, CD32/FcγRIIb, and CD85j and lower levels of CD27, CXCR4, CXCR5, CCR7, and CD62L than conventional MBCs (37, 38, 39, 40, 41) (Table 1). They also upregulated PC-related genes following pathogen exposure in vivo but exhibited reduced responses relative to naïve and conventional MBCs to in vitro stimuli (37, 38, 39, 42). Indeed, despite adopting a transcriptional signature resembling PC, atypical MBCs produced 100-fold lower amounts of Ig when cocultured with autologous cTfh cells (41, 42). This was consistent with low expression of transcripts associated with BCR signaling, and reduced Ca^2+^ flux and phosphorylation of BLNK, SYK, and PLCγ in atypical MBCs compared with naïve and MBCs (38, 40). Malaria-associated atypical MBCs lacked FCRL4 but expressed the related inhibitory molecule FCRL5 (38, 40, 41) (Table 1). In HDs and malaria-infected individuals, atypical B cells expressing the highest levels of FCRL5 also expressed higher levels of CD11c, CD19, CD21, CD22, CD85d, CD85j, and CD95, and lower levels of CD21, CXCR4, and CXCR5, had undergone greater proliferation and SHM in vivo, but had less capacity to produce IgG in vitro compared with FCRL5^−^ atypical MBCs (39, 41, 43). This underscores atypical MBC phenotypic and functional heterogeneity and suggests differential expression of canonical surface receptors reflects graded stages of maturation of these B cells (Fig. 3 B).

Longitudinal assessment of individuals in malaria-endemic areas before, during, and after acute infection showed that *P. falciparum* infection increased levels of serum IFNγ (40). Complementary studies reported IFNγ could induce T-bet and other features of atypical MBCs (↑ FCRL5, CXCR3, CD95) in human naïve B cells stimulated with TLR7/TLR9 ligands and/or BCR agonists in vitro (40, 44). This was replicated when naïve B cells were cocultured with Th1 or cTfh1 cells in an IFNγ-dependent manner (40, 45), inferring a critical role for infection-induced IFNγ in the expansion of atypical MBCs.

#### Other viral infections

Chronic hepatitis virus infection is associated with liver disease, autoimmunity, and lymphoproliferation. Given associations between CD21^lo^ B cells, autoimmunity, and chronic pathogen infection, it is not surprising that CD21^lo^ B cells are also increased in HBV- and HCV-infected individuals (46, 47, 48) (Fig. 2 and Table 1). CD21^lo^ B cells expanded in HCV infection had a CD11c^+^FCRL4^hi^CD19^hi^CD20^hi^CD22^hi^CD27^+^CD72^hi^CD95^hi^ phenotype, expressed increased levels of *SOX5* and *ZEB2* but low levels of *BACH2*, exhibited reduced Ca^2+^ flux, and were hypoproliferative in response to BCR engagement (46, 47, 48) (Table 1). Interestingly, CD21^lo^ B cells in HCV infection were enriched for autoreactive BCRs (46, 47), and increased proportions of these B cells could be reduced following antiviral treatment (46, 47).

Individuals with chronic HBV (cHBV) infection do not generate adequate levels of anti-hepatitis B surface Ag (HBsAg) IgG, the production of which is associated with infection resolution and effective cure of HBV-induced disease (49). Studies by Burton et al. and Salimzadeh and Le Bert et al. addressed this by defining the nature of HBV-specific B cells in infected individuals who either completely resolved infection or developed cHBV infection, as well as in individuals vaccinated against HBV (50, 51). Both studies found that (1) HBV-specific B cells isolated from people with cHBV infection had a markedly reduced capacity to differentiate into plasmablasts producing HBV-specific Ab (10-fold) and cytokine-expressing cells (two- to threefold) relative to those from vaccinated donors, and (2) frequencies of CD21^lo^-type B cells (defined by high-dimensional flow cytometry as CD21^−^CD27^−^) were significantly increased within the population of HBsAg-specific B cells, as well as total B cells, in individuals with cHBV compared with vaccinated HDs (50, 51). Notably, in cHBV infection, enrichment of CD21^lo^ B cell proportions was greater within the Ag-specific B cell subset (∼3–5-fold) compared with total B cells (∼1.5–2.5-fold) (50, 51). Furthermore, CD21^lo^ B cells were detected within intrahepatic lymphocyte populations but at greater frequencies in liver samples obtained from cHBV-infected individuals compared with HDs (50). Analysis of paired blood and liver tissues from cHBV-infected individuals showed that proportions of CD21^lo^ B cells were greater in intrahepatic lymphocytes compared with peripheral blood samples (50, 52). Phenotypically, these B cells resembled CD21^lo^ B cells detected in other disease settings, expressing high levels of CD11c, CXCR3, FCRL5, CD22, BTLA, PD1, and FcγRIIb, and reduced levels of CXCR5 and CD80 (50, 51).

The high level of PD-1 on HBsAg-specific B cells led to question whether PD-1 blockade may enhance or restore the function of these cells, at least in vitro. The ability of HBsAg-specific B cells from vaccinated HDs or cHBV-infected individuals to differentiate into Ab-secreting plasmablasts was enhanced ~2-fold or ∼10-fold, respectively, by the presence of anti-PD-1 mAb (51). However, differentiation of PD-1–treated HBsAg-specific B cells from cHBV-infected individuals remained below that of untreated HBsAg-specific B cells from vaccinated HDs (51). Despite this, these data are consistent with studies of CD21^lo^ B cells in HIV, the function of which could be improved by siRNA-mediated downregulation of inhibitory receptors (36), suggesting avenues to potentially restore functional impairments of CD21^lo^ B cells in different disease settings.

Thus, increased proportions of CD21^lo^CD11c^+^ B cells are significantly associated with chronic pathogen infections characterized by dysfunctional humoral immunity evidenced by hypergammaglobulinemia but impaired pathogen-specific Ab responses. The findings that frequencies of CD21^lo^CD11c^+^ B cells in these infections can be reduced when pathogen load is also reduced suggest that expansion of these B cells is driven by chronic exposure to high antigenic loads.

### IgD*^−^*CD27^−^ DN B cells (and other flavors of “atypical” B cells) in SLE

Flow cytometric analysis of human B cells using mAbs against IgD and CD27 identifies three prominent populations comprising >95% of all peripheral blood B cells in HDs: IgD^+^CD27^−^ transitional/naïve B cells (∼60–75%), IgD^lo/+^CD27^+^ unswitched MBCs (IgM^hi^IgD^lo^, IgM^only^, 10–20%), and IgD^−^CD27^+^ class-switched MBCs (IgG^+^ IgA^+^; 15–30%) (53). Using this approach, Inaki Sanz and colleagues observed that the B cell population lacking both IgD and CD27 (IgD^−^CD27^−^ DN) was significantly expanded in SLE patients (54, 55) (Table 1; and Figs. 1 and 2). DN B cells from HDs and SLE patients exhibited comparable levels of SHM, but less than conventional CD27^+^ MBCs. Interestingly, SLE patients with the highest frequencies of peripheral blood DN B cells had more severe clinical features, such as nephritis and greater titers of anti-double stranded DNA autoAbs (54).

The expanded DN B cell population in SLE was further refined into DN1 (CXCR5^+^CD19^+^CD21^+^CD11c^neg^) and DN2 (CXCR5^neg^CD19^hi^CD21^lo^CD11c^+^) subsets, with each being the dominant DN B cell population in HDs and SLE patients, respectively (56). DN2 cells appear to arise from activated naïve B cells, possibly via an extrafollicular reaction, and acquire expression of the transcription factors T-bet and ZEB2 (56) (Fig. 1). Notably, SLE patients with the highest proportions of DN2 B cells exhibited higher disease activity scores, as well as serum levels of autoAbs, IFNγ, and IFNγ-induced inflammatory mediators such as CXCL10, TNFα, and IL-6 (45, 56). SLE DN2 cells were clonally related to activated naïve B cells and PCs secreting pathogenic autoAbs (56, 57). Consistent with increased Blimp1 and IRF4 expression, DN2 B cells efficiently differentiated into plasmablasts secreting such autoAbs in response to in vitro stimulation with TLR7, IL-21, and IFNγ (56, 57). Thus, in SLE, DN2 B cells are the main population of expanded B cells, appear to be derived from activated naïve B cells, and are precursors of pathogenic PC, which can be induced by exposure to immune complexes comprising autoAbs and DNA/RNA sensors within an IFNγ-rich pro-inflammatory environment (Fig. 1).

Other investigators reported increased frequencies of distinct B cell subsets in SLE - CD19^hi^CD20^hi^ (58), CD11c^+^ (59), or CD24^-^CD20^hi^ (60) - that phenotypically align with DN and CD21^lo^ B cells with respect to increased and decreased expression of activating and inhibitory receptors and transcription factors (Table 1). Expanded CD19^hi^CD20^hi^, CD11c^+^, or CD24^−^CD20^hi^ B cell populations in SLE also correlated with disease manifestations and severity (i.e., increased SLE disease activity [SLEDAI] score, greater incidence of severe neurological and renal pathology over SLE patients who have normal proportions of CD19^hi^CD20^hi^/CD11c^+^/CD24^−^CD20^hi^ B cells), as well as levels of serum autoAbs and circulating PCs (58, 59, 60). CD20^hi^T-bet^+^ B cells were also detectable at increased frequencies in kidneys of patients with lupus nephritis compared with healthy kidneys, and proportions of peripheral blood CD24^−^CD20^hi^ B cells in lupus nephritis patients correlated with CD20^hi^T-bet^+^ B cell proportions in kidneys (60). Furthermore, frequencies of CD20^hi^T-bet^+^ B cells in kidneys positively correlated with the SLEDAI score (60). Thus, enumerating CD24^−^CD20^hi^ (i.e., CD21^lo^ type) B cells in the peripheral blood of SLE patients may predict disease severity, especially renal involvement.

CD24^−^CD20^hi^ B cells in SLE exhibited increased activation of the BCR and PI3 kinase pathways, evidenced from gene set enrichment analysis of RNA sequencing (RNA-seq) data, elevated levels of phospho (p)-BTK, pSYK, and pPLCγ, and increased proportions of p-mTOR^+^ and pS6^+^ B cells compared with classic MBCs (60). Despite evidence of increased basal levels of intracellular signaling activity, and increased expression of some PC transcription factors, CD24^−^CD20^hi^ B cells (i.e., CD21^lo^ type) isolated from SLE patients failed to give rise to appreciable numbers of Ig-secreting cells following in vitro stimulation, in contrast to class-switched MBCs (60). However, low amounts of Ig produced by CD19^hi^CD20^hi^/CD11c^+^/CD24^−^CD20^hi^ B cells were enriched for autoAbs (58, 59, 60), suggesting that despite low production of polyclonal Ig, these cells are precursors of autoreactive plasmablasts (Fig. 1 and Fig. 3 B).

Naïve B cells from HDs and SLE patients differentiated into CD11c^+^T-bet^+^FCRL5^+^ cells in vitro in the presence of activated Th1 cells and/or CD40/BCR/TLR engagement in an IFNγ-dependent manner (45, 56, 59). IFNγ upregulated IL-21R on in vitro–activated B cells, rendering B cells sensitive to IL-21, which enhanced this response by greater than twofold (45, 56, 59). This observation provides a functional explanation for elevated IL-21R expression on CD11c^+^ B cells in SLE patients (59). In vitro induction of T-bet^+^ B cells from HD B cells in response to stimulation with the TLR7 agonist R848 and IFNγ could be attenuated by the mTOR inhibitor rapamycin (60). This is consistent with transcriptomic and biochemical data demonstrating increased activation of the BCR and PI3 kinase pathways, thereby suggesting PI3K signaling may contribute to dysregulated generation of CD21^lo^-type B cells in SLE.

### CD21^lo^ B cells and other autoimmune conditions and pathologies

Due to the association of increased CD21^lo^ B cells in CVID with autoimmune features (i.e., cCVID [10, 11]) and SLE (54, 55, 58, 59, 61), several groups quantified these cells in other systemic autoimmune diseases (Fig. 2). CD21^lo^ B cells were significantly increased in rheumatoid arthritis (RA) (13, 14, 59), Sjogren’s syndrome (13, 62), antiphospholipid and antisynthetase syndromes (13), and multiple sclerosis (MS) (63, 64) (Fig. 2 and Table 1). CD21^lo^ B cells were further increased in Sjogren’s syndrome patients with lymphoproliferation (62); were correlated with biomarkers of joint destruction in RA (65); and were present at even greater proportions in MS patients who subsequently developed more severe disease (64). CD21^lo^ B cells were detected at high frequencies in the cerebrospinal fluid (CSF) of MS patients, exceeding proportions detected in paired peripheral blood samples (CD21^lo^ B cells in CSF from HDs were not determined but would presumably be very low) (63). Interestingly, FCRL4^+^ B cells have been detected in the synovial fluid of RA patients (66, 67). These B cells correlated with inflammation and disease progression, and exhibited features of CD21^lo^ B cells, tissue-like MBCs, and DN B cells, i.e., increased *SOX5*, *TNFSF11* (RANKL), *ITGAX* (CD11c), *MS4A1* (CD20), CD95, CD86, and CCR5; reduced *CR2* (CD21); and greater production of autoreactive Abs relative to synovial fluid FCRL4^−^ B cells (66, 67). Whether production of RANKL by FCRL4^+^ CD21^lo^-type B cells contributes to tissue damage in inflamed joints in RA remains to be determined.

Trisomy 21 causes Down’s syndrome, and affected individuals are susceptible to severe infections and autoimmunity. CD11c^+^T-bet^+^ B cells were found to be significantly increased in peripheral blood of people with trisomy 21, despite B cell lymphopenia (68) (Table 1 and Fig. 2). CD11c^+^T-bet^+^ B cells in trisomy 21 had undergone less SHM than corresponding B cells from HDs, and expressed elevated levels of CD86, CD95, CXCR3, and CCR4, and lower CD21, CXCR5, and CCR7, likely reflective of the inflammatory microenvironment of these individuals (68) (Table 1). Indeed, proportions of CD21^lo^CD11c^+^T-bet^+^ B cells in trisomy 21 correlated with serum levels of inflammatory cytokines, especially IL-6, the sum of cTfh1- and cTfh17-type cells, autoimmune score, and circulating PCs (68).

Similar to cCVID, CD21^lo^ B cells in CVID, SLE, RA, Sjogren’s syndrome, and trisomy 21 were generally hyporesponsive to BCR engagement (14, 62, 63), had a phenotype and/or transcriptome indicative of sustained activation and immune regulation (14), and were enriched for self-reactive BCRs, which often correlated with poor clinical outcomes (10, 14, 62, 68) (Table 1). Thus, CD21^lo^ B cells may produce pathogenic autoAbs characteristic of these autoimmune conditions.

Beyond these conditions, CD21^lo^-type B cells are also increased in individuals who developed chronic graft-vs-host disease (GVHD) following hematopietic stem cell transplantation compared with people who did not develop GVHD (69). While CD21^lo^-type B cells do not appear to be increased in type 1 diabetes, such B cells could be detected and were enriched for BCRs with anti-insulin self-reactivity (70). Interestingly, insulin-binding B cells exhibited transcriptomic and phenotypic features of CD21^lo^ B cells reported in other autoimmune conditions (i.e., increased *TBX21*, *ITGAX*, *SOX5*, *ZEB2*, *FCRL5*, CD21^lo^ CD27^int^; Table 1), but were predominantly unswitched IgM^+^ cells and had undergone minimal SHM (70), suggesting they are derived from extrafollicular/non-GC pathways.

## Potential role of CD21^lo^ B cells as Ag-presenting cells in autoimmune diseases

Many studies have shown that CD21^lo^ B cells isolated from people with autoAb-mediated autoimmune diseases produce autoreactive Ig. This clearly represents one mechanism by which these cells potentially contribute to disease pathogenesis. The elevated expression of costimulatory molecules such as CD80 and CD86, and sustained and even increased HLA-DR expression, on CD21^lo^ B cells together with gene ontology analysis from RNA-seq data, led to the suggestion that these B cells may have a role in Ag presentation (13, 43, 71, 72, 73), reminiscent of early findings reported from studies of analogous B cells in mice (74). In vitro cultures demonstrated that CD21^lo^ B cells -irrespective of whether they were isolated from naïve, IgM^+^, class-switched, or DN MBC subsets - could activate allogeneic naïve and memory CD4^+^ T cells to similar levels as conventional CD21^hi^ B cells present in the same subsets, indicating capacity for Ag presentation (13).

More recently, Younis and colleagues investigated mechanisms underpinning associations between EBV infection and subsequent development of autoimmune conditions. For both SLE and MS, EBV-infected B cells were predominantly CD21^lo^CD27^+^T-bet^+^ cells co-expressing *ZEB2* and transcriptional programs associated with Ag presentation (75, 76). While these B cells were detectable in HDs, they were increased ∼25-fold in SLE or RA (75, 76). EBV nuclear antigen 2 (EBNA2) is a transcription factor encoded by the EBV genome. Strikingly, EBNA2 could bind transcriptional sites regulating expression of *TBX21*, *ZEB2*, *CD27*, and Ag presentation genes (75, 76), suggesting EBV infection itself may accelerate the differentiation of precursor B cells to a pathogenic CD21^lo^CD27^+^ B cell fate in human autoimmunity. Consistent with this, EBV^+^ B cells isolated from SLE or MS patients - but not HDs - expressed BCRs specific for disease-relevant autoantigens (75, 76). Lastly, in an in vitro autologous coculture system, EBV-immortalized B cell lines generated from SLE or MS patients could present autoAg to CD4^+^ T cells, resulting in activation of responding CD4^+^ T cells and concomitant differentiation of EBV^neg^ B cells into autoAb-producing plasmablasts (75, 76). Thus, in SLE and MS, EBV-infected B cells - which are enriched for CD21^lo^CD27^+^ B cells - become rewired to function as Ag-presenting cells for autoreactive CD4^+^ T cells to drive expansion of pathogenic B cells, which become CD21^lo^-type cells or plasmablasts.

## CD21^lo^ B cells and cancer

### Defining tumor-infiltrating B cells

Single-cell RNA-seq (scRNA-seq) technologies have enabled the assessment of primary tissues from a wide range of tumor types to define the nature of B cells present in the tumor microenvironment (TME). Yang et al. analyzed 19 different cancers and identified 20 transcriptomically definable clusters of human B cells. This revealed eight clusters of MBCs, including two subgroups of CD21^lo^-like B cells, defined by the increased expression of *FCRL4*, *FCRL5*, *TBX21* (T-bet), *ITGAX* (CD11c), and *PDCD1* (PD-1) and reduced expression of *CR2* (CD21) (77). Using a similar approach, Ma et al. established an atlas of tumor-infiltrating B cells across 20 different human cancers (78). This identified 15 B cell clusters, including a CD21^lo^-type cluster defined according to the expression of *DUSP4*, *ITGAX* (CD11c), *FCRL5*, and *ZEB2* (78). Further transcriptomic analysis of these TME-associated CD21^lo^-type B cells revealed increased expression of transcription factors *PRDM1*, *IRF4*, *XBP1*, *TOX*, and *TOX2*, surface receptors *PDCD1* (PD-1), *FCRL4*, *CD32A*, *CD32B*, *CD80*, *CD86*, and *CD72*, and the signaling molecules *SYK*, *TLR7*, and *TLR9*, and reduced expression of *CD27*, *CD38*, and *IRF8* in comparison with MBCs (78). High-dimensional flow cytometric assessment of patients with head and neck squamous cell carcinoma (HNSCC), melanoma, or non–small-cell lung carcinoma revealed increases in CD21^−^CD27^−^ DN B cells in patient blood and tumor samples compared with HDs (72). These CD21^lo^-like B cells identified in the TME appeared to be of extrafollicular origin and were phenotypically comparable with those detected in chronic infection (i.e., heightened expression of T-bet, CD85j, and LAIR1) (72). Similar to CD21^lo^ B cell detected in other disease settings, CD21^lo^-type B cells in the TME were hyporesponsive to BCR engagement and—despite increased expression of PC transcription factors—exhibited inefficient differentiation into Ig-secreting cells in vitro, producing lower amounts of total Ig compared with MBCs (72, 78). However, Ig secreted by CD21^lo^-type B cells was enriched for autoreactivity and lacked specificity against tumor-associated Ags (78). Thus, CD21^lo^CD11c^+^T-bet^+^ B cells infiltrate the TME of multiple types of cancers and appear to resemble similar cells present in healthy and disease states.

### Association of CD21^lo^ B cells with cancer outcomes

The studies discussed above also measured correlations between different B cell subsets in TMEs and disease outcomes. Yang et al. found that an enrichment of CD21^lo^-type B cells in the TME correlated with prolonged patient survival across all cancers (77). However, there were some exceptions to this pan-cancer finding, as an increased CD21^lo^-type B cell signature was associated with worse outcomes for patients with HNSCC and colon adenocarcinoma (77). Strikingly, Ma et al. reported increased proportions of TME CD21^lo^-type B cells correlated with poor survival and poor responses to treatment across several cancer types, namely colon and stomach adenocarcinomas, lung cancer, and hepatocellular carcinoma (78). Ruffin et al. also noted that patients with more advanced HNSCC had greater proportions of CD21^lo^-type B cells in TME and peripheral blood than patients with early-stage cancer (72). Despite these juxtaposing results, there is common ground regarding negative associations between increased proportions of CD21^lo^-type B cells in the TME and poor patient outcomes in some specific malignancies (77, 78). Clearly, additional studies need to more clearly define the positive or deleterious impact of CD21^lo^ B cells on cancer outcomes, but the negative correlations are supported by the findings that these B cells isolated from TME produced autoAbs but not antitumor Abs (78).

### Checkpoint inhibitor immunotherapy modulates B cell subsets in cancer patients

Treatment and outcomes for people with cancer have been revolutionized by the development of immune tolerance checkpoint inhibitors (ICIs), mAbs that block the inhibitory effects of CTLA4 and PD-1, thereby unleashing effector functions of various immune cell types, but predominantly CD4^+^ and CD8^+^ T cells (79). Consequently, immunotherapy with anti-PD-1 and/or anti-CTLA4 mAbs is now frequently used to treat myriad cancers. However, as these biological inhibitors essentially work by overriding immune tolerance checkpoints, many immune-related adverse events (iRAEs)—e.g., autoimmune enteropathies and endocrinopathies—frequently occur following anti-PD-1 and/or anti-CTLA4 immunotherapy (79). Initial studies of the effects of ICIs on immune cells primarily focused on T cells (79); however, these also modulate the B cell compartment. An early study of advanced melanoma patients treated with anti-PD-1 mAb, anti-CTLA4 mAb, or both found that combined PD-1/CTLA4 blockade resulted in a significant reduction in total B cell frequencies in peripheral blood that was not observed with anti-PD-1 or anti-CTLA4 mAb alone (80). More impressive though were the significant increases in proportions of CD21^lo^ B cells and plasmablasts. The increase in CD21^lo^ B cells also occurred with anti-CTLA4 monotherapy, indicating PD-1 blockade had a limited effect on regulating CD21^lo^ B cells in this setting (80). Compared with CD21^+^ B cells present in the same individual, the expanded CD21^lo^ B cells after combined ICI treatment were CD40^lo^CXCR4^lo^CXCR5^lo^CD95^hi^PD-1^hi^ and exhibited a transcriptional signature suggestive of B cell activation and IFNγ signaling (80). CD21^lo^ B cells were also enriched for Ki67^+^ cells, indicating the increase in CD21^lo^ B cells likely resulted from de novo ICI-induced proliferation (80). Lastly, the magnitude of the changes in B cell populations following ICI treatment correlated with the likelihood of developing more serious iRAEs (80), while patients with more advanced disease (HNSCC) following anti-PD-1 ICI had greater proportions of circulating CD21^lo^-type B cells prior to commencing immunotherapy than patients who exhibited stable disease (72). These findings suggest that CD21^lo^ B cells may contribute to autoimmune pathology after ICI, consistent with TME CD21^lo^-type B cells producing autoAbs rather than antitumor Abs (78), and/or predict ICI therapeutic outcomes. Thus, monitoring B cell subsets may identify individuals more likely to develop severe iRAEs.

## CD21^lo^ B cells and humoral immunity following natural infection or vaccination

While CD21^lo^ B cells are clearly expanded in various types of immune dysregulation, teleologically it is counterintuitive for the human immune system to carry a cell type that is only associated with disease states and is likely pathogenic. Thus, it would be expected that CD21^lo^ B cells—multiple subsets of which are present in peripheral blood of HDs (Table 1) (43)—have a role in normal immune responses to pathogens or vaccines. This concept has led to several studies assessing the appearance of CD21^lo^ B cells following infection or routine vaccination.

Proportions of peripheral blood B cells with features of CD21^lo^ B cells expanded 2 wk after vaccination with live yellow fever or vaccinia (smallpox) vaccines, reaching a peak after ∼3 wk and declining after 4–5 wk (32). These observations parallel responses to seasonal influenza vaccination, or infection with and/or vaccination against SARS-CoV-2. Specifically, CD21^lo^ B cells comprised 5–10% of flu- or ∼20–60% of SARS-CoV-2 Spike-specific B cells 2–4 wk after vaccination but declined to 3–10% after 6–12 mo, at which time most (60–80%) Ag-specific B cells acquired a phenotype of conventional MBCs (81, 82, 83, 84). In these studies, CD21^lo^ B cells dominating the Ag-specific B cell population during the initial postvaccine/infection time frame had a phenotype that overlapped with B cells expanded in cCVID, autoimmune conditions, and chronic infection, i.e., CD11c^+^CD80^+^CD95^+^ FCRL5^+^CD85j^+^T-bet^hi^Ki67^+^Blimp1^hi^*XBP1*^hi^*BCMA*^hi^IL-6R^hi^CXCR4^−^ CXCR5^−^BACH2^lo^ (81, 82, 83) (Table 1). The decline in CD11c^+^CD21^lo^T-bet^+^ B cells at later times after vaccination may reflect regulation by inhibitory/apoptotic receptors or molecular networks, differentiation into conventional MBCs, or migration from the blood to lymphoid tissues under the influence of CXCR3 ligands. Interestingly, expansion of flu-specific FCRL5^+^CD21^lo^-type B cells was reduced in elderly individuals (aged 66–89 years) compared with younger individuals (aged 18–36 years), and this correlated with poorer Ag-specific Ab responses in the elderly (85), suggesting effective induction of these B cells contributes to better humoral immune protection.

Intriguingly, SARS-CoV-2 infection resulting in severe life-threatening COVID-19 was also associated with the rapid appearance of large proportions of CD21^lo^CD11c^+^T-bet^hi^ B cells in some individuals (86), while individuals with increased basal proportions of CD21^lo^ B cells exhibited poor humoral responses to SARS-CoV-2 mRNA vaccines, evidenced by reduced titers of neutralizing IgG and reduced proportions of specific B cells (73, 87). Indeed, the frequency of CD21^lo^-type B cells negatively correlated with SARS-CoV-2-specific neutralizing Abs and MBCs following vaccination (73). Thus, CD21^lo^CD11c^+^T-bet^+^-type B cells transiently increase during Ag-specific B cell responses but paradoxically can also be associated with productive, weak, or deleterious outcomes depending on context.

## Summary so far

Studies over the past 25 years identified a human B cell subset that could generally be defined as CD21^lo^T-bet^+^CD27^−/+^ expressing high levels of inhibitory and inflammatory chemokine receptors and reduced levels of canonical B cell markers and homeostatic chemokine receptors (Fig. 1 and Table 1). Additional features are enrichment of self-reactive BCRs, hyporesponsiveness to BCR engagement, and predisposition to apoptosis, but also plasmablast differentiation. CD21^lo^T-bet^+^ B cells are increased in immune dysregulatory conditions associated with B cell dysfunction—autoAb production, anergy/exhaustion, reduced participation in GC reactions, impaired humoral immunity—which can manifest as poor responses to pathogens or vaccines but also immune dysregulation. These clinical findings implicate CD21^lo^T-bet^+^ B cells in numerous immune pathologies. Despite this, CD21^lo^T-bet^hi^ B cells may contribute to humoral immunity elicited by specific Ags. However, the relevance of the dominance of these B cells during early phases of Ag-specific B cell responses is enigmatic and incompletely resolved. Thus, it remains challenging to determine how increased proportions of CD21^lo^T-bet^+^ B cells are associated with opposing immune outcomes: immune deficiency, immune dysregulation, autoimmunity, severe/fatal COVID-19, and early or poor responses to vaccination. Similarly, there is profound heterogeneity in phenotypes, transcriptomic signatures, and functions of CD21^lo^ B cells. scRNA-seq and high-dimensional cytometric studies performed on HDs and myriad human diseases frequently identified multiple clusters of CD21^lo^ B cells that differ according to expression patterns of various surface receptors (CD11c, CXCR3, FCRL4, FCRL5) and transcription factors (39, 42, 43, 56, 72, 75, 77, 78, 85, 88, 89). It remains unclear whether this heterogeneity reflects (1) differences in times of analysis of CD21^lo^ B cells with respect to disease onset and severity, infection, or vaccination, (2) the influence of the microenvironment, (3) the precursor cell that committed to the CD21^lo^ B cell fate, (4) the pathway of origin (i.e., via GC or extrafollicular pathways), or (5) maturation of CD21^lo^ B cells along a differentiation spectrum (Fig. 3 B). Thus, much remains to be understood about the physiological role of CD21^lo^T-bet^+^ B cells.

## IEIs as a pathway to understanding the biology of CD21^lo^ B cells

By studying IEIs, we have been able to determine redundant and non-redundant roles of specific genes, proteins, and signaling pathways in the generation and differentiation of distinct human lymphocyte subsets (2, 90, 91). Thus, IEIs may fill some knowledge gaps regarding the requirements for the development and function of CD21^lo^ B cells.

### CD21^lo^ B cells are expanded in IEIs associated with autoimmune features

Quantification of CD21^lo^ B cells in different IEIs revealed increased proportions in patients with immune dysregulation due to loss-of-function (LOF) variants in *CTLA4* (16, 17, 21, 92, 93), *LRBA* (92, 94), *FAS* (CD95), *NFKB1*, *AICDA*, *ADA2* (17), *PDCD1* (encoding PD-1) (95), *STAT5B* (96), or *SOCS1* (97); gain-of-function (GOF) variants in *STAT1* or *STAT3* (17, 18); or hypomorphic variants in *RAG1* or *RAG2* resulting in partial RAG deficiency (98, 99). From this, several hypotheses could be proposed regarding mechanisms regulating CD21^lo^ B cells. First, dysregulated cytokine signaling due to excessive basal or cytokine-induced STAT1 activation may promote CD21^lo^ B cell accumulation in a cell-intrinsic manner in individuals with STAT1 GOF or SOCS1 LOF. Similarly, *STAT3* GOF variants may render B cells intrinsically hyperresponsive to cytokines that ordinarily do not require STAT3 for cytokine-induced generation of CD21^lo^T-bet^+^ B cells. However, this remains to be tested experimentally.

Second, CD21^lo^ B cell expansion may be extrinsically regulated by T cells. Regulatory T cells (Tregs) may restrain CD21^lo^ B cells, thus explaining increased frequencies of CD21^lo^ B cells in IEIs due to variants in *CTLA4*, *LRBA*, or *STAT5B* that affect Treg development or function (100), as well as in melanoma patients treated with anti-CTLA4 mAb ICI treatment (80). Notably, Tregs are significantly contracted and constrained in partial RAG deficiency (98, 99). cTfh1 cells, which produce high amounts of IFNγ (22, 101), may drive expansion of CD21^lo^ B cells in physiological settings (e.g., vaccination), but this is dysregulated in several IEIs and related conditions. Evidence supporting this includes: (1) cTfh1 cells are the prominent cTfh subset that emerges following influenza vaccination, and correlate with flu-specific CD21^lo^CD27^+^ B cells and influenza-specific IgG (84); (2) cTfh1 cells are aberrantly increased in STAT1 GOF (21, 101), STAT3 GOF (18), and LRBA or CTLA4 deficiency (92); and (3) increases in CD21^lo^ B cells in partial RAG deficiency correlated with serum levels of IFNγ-induced CXCL9 and Th1 cells (98, 99). This is consistent with cCVID, or HIV or malaria infection, where frequencies of both CD21^lo^ B and cTfh1 cells are increased and are associated with IFN-γ signatures in CD21^lo^ B cells (22, 23, 29, 33, 40, 89, 102).

Third, pathogenic variants affecting regulatory receptors highly expressed on CD21^lo^ B cells may contribute to their expansion in FAS (17) or PD1 deficiency (95). Curiously, responses of some cancer patients to ICIs inversely correlated with proportions of CD21^lo^ B cells; i.e., these B cells were increased in cancer patients who did not respond to anti-PD-1 mAb treatment compared with HDs and patients who did respond (72, 103). Similarly, changes in B cell frequencies, including an expansion of CD21^lo^ B cells, following ICI therapy predicted poorer patient outcomes (80). Whether expansion of CD21^lo^ B cells in patients treated with ICIs directly contributes to poor responses to cancer immunotherapy is unknown. However, it is not an unreasonable extrapolation given the association of increased CD21^lo^ B cells and immune dysregulation in cCVID, autoimmunity, chronic infection, and various IEIs, and the poor ability of these B cells to produce functional Abs.

### IEIs reveal what is, and what is not, required for generating CD21^lo^ B cells

Human CD21^lo^T-bet^+^ B cells exhibit an IFNγ-gene signature ex vivo (15, 23, 56, 89), suggesting a role for IFNγ in their generation. Consistent with this, stimulation of naïve B cells through the BCR, CD40, TLRs, and IFNγR can give rise to T-bet^+^CD21^lo^-type B cells in vitro (17, 19, 35, 40, 44, 45). Naïve B cells also differentiate into these B cells in vitro in response to CD40L/anti-Ig/IL-21 (59), while IL-27 has a similar effect as IFNγ at inducing T-bet^+^CXCR3^+^ B cells from anti-Ig/CpG-primed B cells (19). While these studies provide a foundation for delineating fundamental requirements for generating, and the further differentiation of, CD21^lo^ B cells, the ultimate test of what is and is not redundant can be gleaned from IEIs.

This was recently addressed by quantifying CD21^lo^CD19^hi^ B cells in patients with a wide array of IEIs. CD21^lo^CD19^hi^ B cell proportions were unaffected by LOF variants in *CD19*, *NFKB2*, *IL2RG*, *IL12RB1*, *IL6ST*, *TNFRSF13B* (TACI), or *STAT3* (Fig. 3 A) (17, 19). These findings are not particularly surprising as these pathways have generally not been implicated in human CD21^lo^T-bet^+^ B cell biology. This also indicates that the accumulation of CD21^lo^T-bet^+^ B cells in STAT3 GOF (17, 18) probably reflects aberrant STAT3 function rather than exaggeration of a physiological role of STAT3 in generating CD21^lo^T-bet^+^ B cells, in which case a reduction in these cells may have been expected in STAT3 LOF (Fig. 3 A).

Strikingly, proportions of CD21^lo^T-bet^+^ B cells were completely unaffected by autosomal recessive IRAK4 deficiency (Fig. 3 A) or MyD88 deficiency (17, 19) despite the requirement for MyD88 in generating age-associated B cells in mice (104). Thus, human CD21^lo^T-bet^+^ B cells develop independently of most TLR signaling pathways. Whether TLR3, which does not require MyD88/IRAK4 for signaling (105), has a role in generating human CD21^lo^T-bet^+^ B cells has not been reported, but pathogenic variants in *TLR3* have (105, 106, 107), so analysis of these individuals could answer this question. CD21^lo^T-bet^+^ B cells were also unaffected by autosomal recessive CD4 deficiency (20) (Fig. 3 B). This is a recently described IEI where affected individuals have pathogenic variants in *CD4*, preventing expression and function of the CD4 protein (20). CD4-deficient individuals lack detectable CD3^+^CD4^+^ T cells but generate a population of CD3^+^CD8^−^ T cells that phenotypically and functionally resemble classical CD4^+^ T cells (20). Thus, conventional interactions between B cells and CD4^+^ T cells via MHC class II and CD4 are redundant for generating CD21^lo^ B cells.

CD21^lo^ B cells were reduced in patients with IEIs that compromise provision of CD4^+^ T cell help (*CD40LG*, *IL21R*) (Fig. 3 A) (17), consistent with the demonstration that CD40L and IL-21, together with other inputs, converge to generate T-bet^+^CD21^lo^-type B cells in vitro (17, 40, 44, 45). While these findings from IEIs support a key role of CD4^+^ T cells in the generation and maintenance of T-bet^+^CD21^lo^-type B cells, this could be explored further, mainly because one of the early studies of CD21^lo^ B cells came from HIV infection, where these B cells are expanded, yet HIV-infected individuals have CD4^+^ T cell lymphopenia (29, 30, 31, 33). This could be done by monitoring the dynamics and kinetics of decline in CD4^+^ T cells and expansion of CD21^lo^ B cell following HIV infection, as well as assessing CD21^lo^ B cells in IEIs affecting MHC class II expression, which results in monogenic CD4 T cell deficiency (108, 109).

CD21^lo^CD19^hi^ B cells tended to be lower but still within the reference range in individuals with *JAK1*, *IFNGR*, *IL27R*, or *STAT1* LOF variants (17, 19) (Fig. 3 A). However, CD21^lo^CD19^hi^ B cells were greatly reduced in the only known individual with complete T-bet deficiency (19, 110) (Fig. 3 A). Coincidentally, while proportions of cTfh cells were not affected by T-bet deficiency, the CXCR3^+^ cTfh1 subset was dramatically reduced, as was IFNγ production, compared with HDs (19, 110). Further analysis revealed that while low but detectable frequencies of CD21^lo^CD19^hi^ B cells could be generated despite impaired IFNγR/JAK1/STAT1 signaling, expression of CXCR3 and T-bet itself was reduced by STAT1 deficiency, while upregulation of CD11c, CXCR3, FCRL5, and T-bet was abolished by T-bet deficiency (19) (Fig. 3 A). Thus, while the initial stages of differentiation to a CD21^lo^T-bet^+^ B cell fate is independent of STAT1/T-bet signaling, subsequent progression to CD21^lo^T-bet^+^CXCR3^+^CD11c^hi^FCRL5^+^ B cells requires STAT1/T-bet downstream of IFNγ, and possibly IL-27 (Fig. 3 B). The finding that T-bet–deficient or STAT1-deficient naïve B cells underwent initial stages of differentiation in vitro (upregulation of T-bet) but were unable to acquire additional features of CD21^lo^ B cells—upregulated expression of CXCR3, FCRL5—indicates the STAT1/T-bet axis functions intrinsically in B cells to generate CD21^lo^ B cells. Combined, these studies established a fundamental B cell–intrinsic role for T-bet—induced downstream of IFNγ—in generating CD21^lo^T-bet^+^ B cells (Fig. 3, A and B).

ZEB2 is another transcription factor highly expressed by CD21^lo^T-bet^+^ B cells (56, 69, 77, 78, 111). *ZEB2* haploinsufficiency causes Mowat–Wilson syndrome (MWS), which is characterized by a distinct dysmorphic appearance, developmental delay, and intellectual disability (112). A requirement for ZEB2 in generating human CD21^lo^-type B cells was recently concluded from two studies that found proportions of CD19^+^IgD^−^CD27^−^CXCR5^−^ (111) or CD19^hi^CD11c^+^ (113) B cells were reduced 1.5–3-fold in individuals with MWS compared with HDs. Thus, ZEB2 may also be critical for generating human CD21^lo^T-bet^+^ B cells. There are several caveats to these studies of human ZEB2 deficiency. First, the reduction in CD19^+^IgD^−^CD27^−^CXCR5^−^ B cells or CD19^hi^CD11c^+^ B cells was variable and incomplete (111, 113). This may reflect MWS patients being heterozygous for the *ZEB2* variant (112), in which one wild-type allele remains functional. It is therefore possible that the reduction in CD21^lo^ B cells may be greater in the setting of complete ZEB2 LOF, as reported for mice conditionally deficient for *Zeb2* in B cells (111, 113). Second, both studies selectively quantified B cell populations that arguably correspond to more differentiated subsets of CD21^lo^-type B cells (111, 113). As not all CD21^lo^ B cells have undergone Ig class switching or acquired CD11c expression (13, 19, 43), it remains unknown at which differentiation stage ZEB2 exerts a transcriptional effect on CD21^lo^ B cells. This of course can be determined by performing more in-depth high-dimensional immune phenotyping of B cells in MWS patients.

Beyond T-bet itself, many other molecules that appear to be critical for generating CD21^lo^T-bet^+^ B cells—e.g., TLR and STAT1 pathways—are partially redundant. The large number of IEIs where CD21^lo^T-bet^+^ B cell frequencies are not affected argues that multiple compensatory pathways exist to ensure these cells can be generated even when a likely dominant pathway (e.g., TLR) is compromised by disease-causing variants. By extension, this argues for an important role for CD21^lo^T-bet^+^ B cells in human immune function and regulation. However, the precise role of these B cells remains a mystery! This is exemplified by individuals with T-bet or ZEB2 deficiency not appearing to manifest clinical features characteristic of defective humoral immunity.

### IEIs can inform treatments and mechanisms of diseases associated with CD21^lo^T-bet^+^ B cells

The beauty of IEIs is that the genetic cause, and thus mechanism(s) of disease pathogenesis, is often known (106). This enables implementation of therapies targeting specific pathways to treat some IEIs (114). Expansion of CD21^lo^T-bet^+^ B cells in monogenic immune dysregulation provides exciting opportunities to further explore the contribution of these B cells to disease pathogenesis and clinical manifestations. Thus, longitudinal immune monitoring of IEI patients will establish whether improvements in disease following commencement of specific targeted therapies are associated with reductions in CD21^lo^T-bet^+^ B cells and other biomarkers or putative disease drivers such as cTfh1 cells and IFNγ signatures. For example, JAK inhibitors are efficacious in treating STAT1 GOF, STAT3 GOF, or SOCS1 deficiency (97, 115, 116). Similarly, abatacept—a CTLA4–human IgG fusion protein—is very effective in treating CTLA4 deficiency and LRBA deficiency (92, 117), which result from defective Tregs. Quantifying CD21^lo^T-bet^+^ B cells in these IEIs, together with other clinical and laboratory readouts (92), before and after treatment will significantly expand our understanding of molecular and cellular networks that underpin putative pathogenicity and autoreactivity of CD21^lo^T-bet^+^ B cells in many human dyscrasias.

Novel IEIs also provide patient cohorts from whom valuable information regarding CD21^lo^T-bet^+^ B cells may be obtained. SYK is highly expressed and phosphorylated in resting CD21^lo^T-bet^+^ B cells (118, 119). SYK GOF causes a multiorgan inflammatory disease, and a mouse model harboring a pathogenic human *SYK* variant phenocopied human immune pathology (120). Importantly, treatment with an experimental SYK inhibitor partially resolved disease in mice (120). Although CD21^lo^ B cells were not assessed in SYK GOF humans or mice (120), this may be a model to test the effects of heightened SYK activation on the differentiation and pathogenicity of CD21^lo^ B cells, and the impact of SYK inhibition on these processes. Attenuation of BTK function downstream of SYK by BTK inhibitors may also be an attractive approach to modulate CD21^lo^ B cells in human diseases, while avoiding complete B cell depletion routinely achieved by anti-CD20 mAb therapies. Lastly, while complete deficiency of MYD88 or IRAK4 did not affect human CD21^lo^ B cells (17, 19), aberrant TLR7 signaling appears to contribute to CD21^lo^ B cell dysregulation in SLE in humans (56) and mouse models (104). Hemizygous *TLR7* LOF or GOF variants have recently been found to cause severe COVID-19 (121) or monogenic SLE (122), respectively. Studying inborn errors affecting TLR7 signaling has the potential to reveal additional features of CD21^lo^ B cells.

## Conclusion

Major advances in our understanding of B cell development and differentiation in general have been achieved by multipronged approaches of studying the molecular and cellular immunology of B cells in settings of healthy humans and common diseases, mice and murine models of human disease, and IEIs, which have the benefit of being monogenic experiments of nature. The application of these orthogonal approaches to studying enigmatic CD21^lo^ B cells has yielded a treasure trove of novel insights into the ontogeny, differentiation, and putative functions—physiological and pathological—of this B cell subset. However, fundamental questions remain, especially regarding the contribution of CD21^lo^ B cells in immune health and immune regulation. Continued analysis of these fascinating B cells in known and novel IEIs will bridge these knowledge gaps and provide key insights into modulating these cells to either treat immune diseases or harness their protective function. Let’s see what the next 25 years of research into CD21^lo^ B cells reaps!
